# Isolation of non-albicans Candida and associated factors among symptomatic women presenting with abnormal vaginal discharge at Mubende Regional Referral Hospital, Central Uganda: A cross-sectional study

**DOI:** 10.1371/journal.pgph.0006620

**Published:** 2026-06-26

**Authors:** Suleiman Ameir Idrissa, Marie Pascaline Sabine Ishimwe, Musa Kasujja, Maxwell Okello, Theodore Nteziyaremye, Sara Liban, Ahmed Kiswezi Kazigo, Olabisi Surat Akib, Theoneste Hakizimana

**Affiliations:** 1 Department of Obstetrics and Gynecology, Kampala International University, Ishaka, Uganda; 2 Department of Pediatrics and Child Health, Kampala International University, Ishaka, Uganda; 3 Department of Sciences, University of Rwanda, Kigali, Rwanda; 4 Department of Surgery, Kampala International University, Ishaka, Uganda; 5 Department of Pathology, Kampala International University, Ishaka, Uganda; PLOS: Public Library of Science, UNITED STATES OF AMERICA

## Abstract

Vulvovaginal infections contribute substantially to women’s reproductive morbidity, yet little is known about the determinants of *non-albicans Candida* (NAC) in sub-Saharan Africa. Understanding population-level drivers is critical for prevention and control. We conducted a hospital-based analytic cross-sectional study among 314 symptomatic women aged 18–49 years presenting with abnormal vaginal discharge at the gynecologic clinic of Mubende Regional Referral Hospital, Central Uganda, between 1^st^ May 2025 and 1^st^ August 2025. High vaginal swabs were cultured and species identified using CHROM agar. Sociodemographic, behavioral, and clinical data were collected through structured interviews. Logistic regression identified determinants of NAC infection. The prevalence of NAC infection was 23.6% (74/314; 95% CI: 19.0–28.8). The most frequently isolated species were *C. glabrata* (41.9%) and *C. tropicalis* (29.7%). Key determinants included: having ≥2 sexual partners (aOR 9.41, 95% CI: 4.59–19.31), ≥ 3 vaginal infections in the past year (aOR 6.43, 95% CI: 3.21–12.89), HIV-positive status (aOR 5.37, 95% CI: 2.51–11.49), contraceptive use (aOR 2.70, 95% CI: 1.39–5.24), vaginal douching (aOR 2.25, 95% CI: 1.15–4.41), and recent antibiotic use (aOR 2.09, 95% CI: 1.06–4.11). NAC infections are highly prevalent among women with abnormal vaginal discharge in Uganda, driven by modifiable behavioral and clinical factors. Public health interventions should prioritize sexual health education, discourage vaginal douching, promote rational antibiotic use, and integrate screening and counselling for high-risk women, especially those with HIV. Interpretation should be cautious because alternative etiologies of vaginal discharge were not excluded and confirmatory species identification was not performed.

## Introduction

Vulvovaginal candidiasis (VVC) remains one of the most common gynecological conditions globally, affecting up to 75% of women at least once in their lifetime and contributing significantly to reproductive morbidity and healthcare costs [1]. While *Candida albicans* has traditionally been the leading pathogen, there is growing recognition of the increasing role of *non-albicans Candida* (NAC) species, particularly C. glabrata, C. krusei, and C. tropicalis [2–4].

NAC infections present unique clinical and public health challenges. They are often associated with recurrent or persistent vaginitis, tend to cause milder but harder-to-diagnose symptoms, and display reduced susceptibility or intrinsic resistance to commonly used azole antifungals, leading to higher rates of treatment failure [5,6]. Contributing risk factors include HIV infection, diabetes mellitus, contraceptive use, prior antibiotic or steroid therapy, advanced age, vaginal douching, and multiple sexual partnerships [7,8].

Epidemiological studies demonstrate wide geographical variation in the prevalence of NAC, ranging from below 15% in Rwanda and Namibia to over 60% in India and Ghana [9–11]. In East Africa, limited studies suggest that NAC infections are an emerging concern, with prevalence rates between 7.9% and 50.8% reported among women in Uganda [12–14]. However, there is a paucity of data on the determinants of NAC infections in Uganda, despite the high burden of HIV, widespread use of antibiotics, and other behavioral and socioeconomic risk factors that may drive susceptibility.

Addressing this evidence gap is critical for guiding targeted interventions, informing empirical treatment, and shaping antifungal stewardship policies in low-resource settings. Therefore, this study was conducted to determine the prevalence of NAC and describe the factors associated with its occurrence among women with abnormal vaginal discharge attending the Gynecologic clinic at Mubende Regional Referral Hospital in Uganda.

## Materials and methods

### Study design and settings

This was a hospital-based, analytic cross-sectional study conducted at the Gynecologic Clinic of Mubende Regional Referral Hospital (MRRH), Uganda from 1^st^ may 2025–1^st^ August 2025. The study focused on assessing the prevalence and determinants of *non-albicans Candida* (NAC) infections among women presenting with abnormal vaginal discharge. Mubende Regional Referral Hospital is a 400-bed facility located in the central business district of Mubende town, approximately 150 km west of Mulago National Referral Hospital and 148 km east of Fort Portal Regional Referral Hospital. The hospital serves as a key referral center for surrounding districts, including Kyankwanzi, Mityana, Kiboga, and Mubende. On average, the Gynecologic Clinic attends to 250–300 patients monthly, including a significant number of women with abnormal vaginal discharge. The hospital’s geographical coordinates are latitude 0.567496 and longitude 31.393041 (0°34’03.0“N, 31°23’35.0”E).

### Study population

The study population comprised women aged 18–49 years who presented with abnormal vaginal discharge at the Gynecologic Clinic of Mubende Regional Referral Hospital during the study period. Eligible participants were those who provided written informed consent. Women who were currently receiving or had recently completed a course of antifungal therapy were excluded. Women with other sexually transmitted or non-fungal causes of vaginal discharge were not specifically excluded through laboratory testing because testing for bacterial vaginosis, trichomoniasis, gonorrhea, chlamydia, vaginal pH, and wet mount/KOH microscopy was not performed as part of the study protocol.

### Sample size determination

Sample size was calculated separately for the two study objectives. For objective 1 (prevalence of *non-albicans Candida*), the Kish-Leslie formula for a single proportion was used with p = 19.84% from a previous Ugandan study [15], yielding n = 244. For objective 2 (factors associated with *non-albicans Candida*), OpenEpi was used to estimate the sample size for comparing two proportions based on vaginal douching as the main exposure [16], yielding n = 141. We adopted the larger minimum sample size (244), added 10% for possible non-response, and obtained a minimum required sample of 268. Consecutive enrollment over the full study period yielded 314 participants, which improved estimate precision and statistical power beyond the minimum requirement.

### Sampling technique

Participants were recruited consecutively from women attending the Gynecologic Clinic at Mubende Regional Referral Hospital (MRRH) until the target sample size of 314 was attained. Eligibility was limited to women aged 18–49 years presenting with abnormal vaginal discharge who provided written informed consent. Women currently on antifungal treatment or those who had recently completed such therapy were not included. Enrollment was carried out without consideration of ethnicity, religion, or other socio-demographic characteristics.

### Data collection procedure

Women who met the inclusion criteria at Mubende Regional Referral Hospital (MRRH) were approached consecutively in the Gynecologic clinic. After explaining the study and confirming understanding, written informed consent was obtained. A structured, interviewer-administered questionnaire was then completed in either English or the local language to capture socio-demographic, behavioral, medical, and gynecological information such as age, parity, contraceptive use, history of vaginal infections, and prior antifungal therapy.

Following completion of the questionnaire, high vaginal swabs were collected under aseptic conditions by trained clinicians. Swabs were immediately placed in sterile, labeled containers and transported to the microbiology laboratory for culture and identification of *non-albicans Candida* species. Samples from women who declined participation were excluded.

### Specimen collection and candida species identification

High vaginal swabs (HVS) were collected under sterile conditions by trained healthcare professionals at the Gynecologic Clinic after obtaining informed consent. Each specimen was labelled with a unique identification code and transported in a non-media specimen container within 5–10 minutes to the certified microbiology laboratory at Mubende Regional Referral Hospital (MRRH).

Swabs were first cultured on Sabouraud Dextrose Agar (SDA) for primary fungal isolation. Colonies suggestive of Candida were subjected to the germ tube test for presumptive identification of *Candida albicans*. All isolates were then sub-cultured on CHROMagar Candida to enable species-level differentiation, with particular attention given to detecting non-albicans Candida (NAC) species. Species identification in this study was phenotypic and presumptive, based on germ tube testing and CHROMagar Candida. These methods are useful for preliminary differentiation but cannot reliably distinguish all closely related non-albicans Candida species, detect emerging species, or replace biochemical or molecular confirmation.

### Study variables

The independent variables included sociodemographic, behavioral, and clinical factors that might be associated with presumptive non-albicans Candida isolation among women with abnormal vaginal discharge. These included age, residence, marital status, education level, and occupation; smoking, number of sexual partners, vaginal douching, and contraceptive use; and clinical characteristics such as HIV status, hypertension, diabetes mellitus, menopausal status, history of vaginal infections in the previous 12 months, recent antibiotic use, and prior treatment for vaginal infection. Pregnancy status and body mass index were not collected in this study and could therefore not be assessed as possible determinants.

The dependent variable was presumptive non-albicans Candida isolation, defined as isolation of any Candida species other than Candida albicans using CHROMagar Candida based on colony morphology and color. Symptomatic women were defined as women aged 18–49 years who presented to the gynecologic clinic with self-reported abnormal vaginal discharge

### Data quality control

To ensure the reliability and credibility of findings on factors associated with *non-albicans Candida* (NAC) infections, strict adherence to inclusion and exclusion criteria was observed. The structured questionnaire was made available in English and the local language for participants with limited literacy. Prior to the main study, the questionnaire was pre-tested on 10% of the target sample at Mubende Regional Referral Hospital, and necessary adjustments were made to enhance clarity and consistency. The principal investigator trained the research assistant and supervised all data collection to ensure correct administration of the questionnaire and compliance with ethical standards.

High vaginal swabs were obtained under sterile conditions by trained personnel, labelled with unique participant codes, and transported promptly to the Mubende certified microbiology laboratory. Ten percent of samples were randomly re-examined at an external reference laboratory to confirm identification accuracy. Data entries were reviewed daily for completeness and accuracy, and all records were securely stored to maintain integrity throughout the study.

To ensure reproducibility of laboratory findings, a randomly selected subset of Candida-positive specimens was rechecked at Orbit Laboratory using the same phenotypic procedures employed at Mubende Regional Referral Hospital. Agreement between the two laboratories was assessed using Cohen’s kappa statistic, and perfect agreement was observed (κ = 1.00), indicating 100% concordance

### Data management and analysis

Data collected were initially coded and entered into Microsoft Excel before being exported to STATA version 14.2 for statistical analysis. Descriptive statistics were used to summarize participants’ Sociodemographic and medical characteristics, with results presented as frequencies, percentages, means, medians, and measures of dispersion such as standard deviations and interquartile ranges.

The prevalence of *non-albicans Candida* (NAC) infection was calculated as the proportion of women testing positive for NAC among all study participants and presented using pie charts. Associations between potential risk factors and NAC infection were evaluated using bivariate analysis (chi-square test for categorical variables and t-test for continuous variables where appropriate). Variables with a p-value <0.2 and biologically plausible were entered into a multivariate logistic regression model. Variables with a p-value <0.05 in the final model were considered statistically significant. Adjusted odds ratios (aOR) with corresponding 95% confidence intervals were reported to quantify the strength of associations.

### Human ethics and consent to participate

Ethical approval for this study was obtained from the Kampala International University Research Ethics Committee under approval number KIU-2025–851. Administrative permission to conduct the study was granted by Mubende Regional Referral Hospital, and the study was registered with the Uganda National Council for Science and Technology (UNCST). Written informed consent was obtained from all participants before enrollment after explanation of the study in a language they understood. Participation was voluntary, and all participants were aged 18 years or older and provided consent personally. Confidentiality was ensured through the use of unique study codes and secure storage of questionnaires, laboratory records, and related study documents. The study adhered to the principles of the Declaration of Helsinki.

## Results

### Characteristics of the study participants

A total of 334 women were approached for recruitment, of whom 325 satisfied the eligibility criteria. Nine women were excluded, while 11 declined participations. Ultimately, 314 consented and were enrolled in the study. Of the 314 enrolled women, most were aged 20–29 years (36.9%), resided in urban areas (54.1%), had primary education (41.7%), and were unemployed (62.4%). Prior vaginal infections in the previous 12 months were reported by 73.9%, recent antibiotic use by 52.2%, and vaginal douching by 61.5% of participants. Detailed participant characteristics are presented in Table 1.

**Table 1:** Sociodemographic and clinical characteristics of study participants (N = 314).

Characteristic	Frequency (n)	Percentage (%)
**Age (years)**		
<20	27	8.6
20-29	116	36.9
30-39	102	32.5
>=40	69	22.0
**Residence**		
Rural	144	45.9
Urban	170	54.1
**Occupation**		
Unemployed	196	62.4
Self-employed	102	32.5
Formal Employee	16	5.1
**Education Level**		
None	38	12.1
Primary	131	41.7
Secondary	96	30.6
Tertiary	49	15.6
**Marital Status**		
Single	146	46.5
Married	168	53.5
**Cigarette Smoking Status**		
Non-smoker	289	92.0
Smoker	25	8.0
**HIV Positive Status**		
No	256	81.5
Yes	58	18.5
**Hypertension**		
No	285	90.8
Yes	29	9.2
**Diabetes Mellitus**		
No	291	92.7
Yes	23	7.3
**History of Vaginal Infection (Last 12 months)**		
0	82	26.1
1-2	155	49.4
>=3	77	24.5
**Recent Antibiotic Use**		
No	150	47.8
Yes	164	52.2
**Treatments for Vaginal infections (in the last 12 months)**		
None	142	45.2
Once	69	22.0
At least twice	103	32.8
**No. of Sexual Partners**		
1	272	86.6
2+	42	13.4
**Vaginal Douching**		
No	121	38.5
Yes	193	61.5
**Contraceptive Use**		
No	198	63.1
Yes	116	36.9
**Menopause Status**		
Not yet	250	79.6
Present	64	20.4

### Prevalence of *Non-albicans Candida* infection

**Fig 1:**
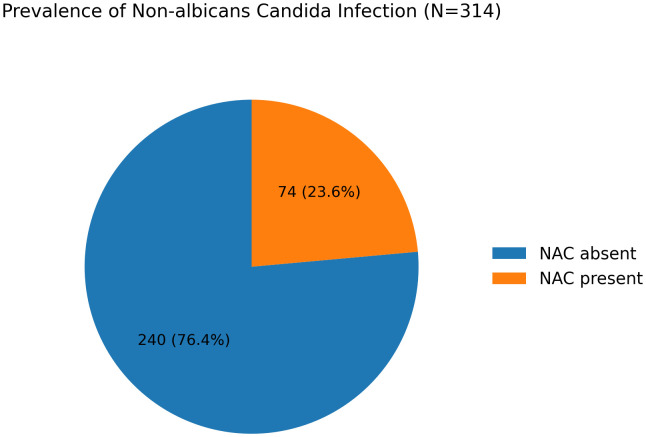
Prevalence of Non-albicans Candida (NAC) Infection among symptomatic women attending Mubende Regional Referral Hospital (N = 314).

Of the 314 women who participated in the study, 74 tested positive for non-albicans Candida, giving an overall prevalence of 23.6% (95% CI: 19.0%–28.7%). The remaining 240 women (76.4%) did not have non-albicans Candida isolated. The distribution is illustrated in Fig 1.

### Factors associated with *Non-albicans Candida* infection

In the bivariable analysis, several factors met the pre-specified threshold of p < 0.20 and were therefore considered for inclusion in the multivariable model. As shown in Table 2, these included HIV-positive status, diabetes mellitus, a history of three or more vaginal infections in the past year, having two or more sexual partners, vaginal douching, contraceptive use, and having received treatment for vaginal infection at least twice in the previous year.

**Table 2:** Bivariate analysis of factors associated with NAC infection (N = 314).

Category	NAC Absent n (%)	NAC Present n (%)	COR (95% CI)	P-value
**Age (years)**				
<20	20 (8.3%)	7 (9.5%)	1.00	
20-29	82 (34.2%)	34 (45.9%)	1.18 (0.46 - 3.06)	0.726
30-39	84 (35.0%)	18 (24.3%)	0.61 (0.23 - 1.66)	0.336
≥40	54 (22.5%)	15 (20.3%)	0.79 (0.28 - 2.23)	0.661
**Residence**				
Urban	131 (54.6%)	39 (52.7%)	1.00	
Rural	109 (45.4%)	35 (47.3%)	1.08 (0.64 - 1.82)	0.777
**Occupation**				
Formal Employee	12 (5.0%)	4 (5.4%)	1.00	
Unemployed	149 (62.1%)	47 (63.5%)	0.95 (0.29 - 3.07)	0.927
Self-employed	79 (32.9%)	23 (31.1%)	0.87 (0.26 - 2.97)	0.828
**Smoking**				
Non-smoker	223 (92.9%)	66 (89.2%)	1.00	
Smoker	17 (7.1%)	8 (10.8%)	1.59 (0.66 - 3.85)	0.304
**Education Level**				
Tertiary	41 (17.1%)	8 (10.8%)	1.00	
None	30 (12.5%)	8 (10.8%)	1.37 (0.46 - 4.05)	0.573
Primary	94 (39.2%)	37 (50.0%)	2.02 (0.86 - 4.71)	**0.105**
Secondary	75 (31.3%)	21 (28.4%)	1.44 (0.58 - 3.53)	0.431
**Marital Status**				
Single	109 (45.4%)	37 (50.0%)	1.00	
Married	131 (54.6%)	37 (50.0%)	0.83 (0.49 - 1.40)	0.490
**HIV Positive**				
No	208 (86.7%)	48 (64.9%)	1.00	
Yes	32 (13.3%)	26 (35.1%)	3.52 (1.92 - 6.45)	**<0.001**
**Hypertension**				
No	217 (90.4%)	68 (91.9%)	1.00	
Yes	23 (9.6%)	6 (8.1%)	0.83 (0.33 - 2.13)	0.702
**Diabetes Mellitus**				
No	225 (93.8%)	66 (89.2%)	1.00	
Yes	15 (6.3%)	8 (10.8%)	1.82 (0.74 - 4.48)	**0.193**
**Prev. Infections (12 months)**				
0	73 (30.4%)	9 (12.2%)	1.00	
1-2	124 (51.7%)	31 (41.9%)	2.03 (0.91 - 4.50)	**0.082**
≥3	43 (17.9%)	34 (45.9%)	6.41 (2.81 - 14.64)	**<0.001**
**Recent Antibiotic Use**				
No	121 (50.4%)	29 (39.2%)	1.00	
Yes	119 (49.6%)	45 (60.8%)	1.58 (0.93 - 2.68)	**0.092**
**Treatments for Vag. Inf.**				
None	119 (49.6%)	23 (31.1%)	1.00	
Once	53 (22.1%)	16 (21.6%)	1.56 (0.76 - 3.19)	0.222
At least twice	68 (28.3%)	35 (47.3%)	2.66 (1.45 - 4.87)	**0.001**
**No. of Sexual Partners**				
1	222 (92.5%)	50 (67.6%)	1.00	
2+	18 (7.5%)	24 (32.4%)	5.92 (2.99 - 11.73)	**<0.001**
**Vaginal Douching**				
No	103 (42.9%)	18 (24.3%)	1.00	
Yes	137 (57.1%)	56 (75.7%)	2.34 (1.30 - 4.22)	**0.005**
**Contraceptive Use**				
No	163 (67.9%)	35 (47.3%)	1.00	
Yes	77 (32.1%)	39 (52.7%)	2.36 (1.39 - 4.01)	**0.001**
**Menopause Status**				
Not yet	191 (79.6%)	59 (79.7%)	1.00	
Present	49 (20.4%)	15 (20.3%)	0.99 (0.52 - 1.89)	0.978

cOR: Crude Odds Ratio; CI: Confidence Interval. Variables with p < 0.20 in bivariable analysis and/or strong biological plausibility were considered for multivariable modeling.

In the multivariable logistic regression model (Table 3), six factors remained independently associated with presumptive non-albicans Candida infection after adjustment for other variables. Having two or more sexual partners showed the strongest association, with more than nine-fold higher odds of NAC (aOR = 9.41, 95% CI: 4.01–22.11). HIV-positive status was associated with more than five-fold higher odds (aOR = 5.37, 95% CI: 2.48–11.66), while a history of three or more vaginal infections in the past year was associated with a 6.43-fold increase in odds (aOR = 6.43, 95% CI: 2.11–19.62). Contraceptive use (aOR = 2.70, 95% CI: 1.42–5.13), vaginal douching (aOR = 2.25, 95% CI: 1.10–4.64), and recent antibiotic use (aOR = 2.09, 95% CI: 1.06–4.11) were also independently associated with NAC.

**Table 3:** Multivariate logistic regression of factors associated with NAC infection (N = 314).

Factors	cOR (95% CI) p-value	aOR (95% CI)	p-value
**No. of Sexual Partners**			
1	1.00	1.00	–
2+	5.92 (2.99 - 11.73) < 0.001	9.41 (4.01 - 22.11)	**<0.001**
**Prev. Vaginal Infections**			
0	1.00	1.00	–
1-2	2.03 (0.91 - 4.50) 0.082	1.77 (0.65 - 4.85)	0.265
≥3	6.41 (2.81 - 14.64) < 0.001	6.43 (2.11 - 19.62)	**0.001**
**HIV Positive**			
No	1.00	1.00	–
Yes	3.52 (1.92 - 6.45) < 0.001	5.37 (2.48 - 11.66)	**<0.001**
**Contraceptive Use**			
No	1.00	1.00	–
Yes	2.36 (1.39 - 4.01) 0.001	2.70 (1.42 - 5.13)	**0.003**
**Vaginal Douching**			
No	1.00	1.00	–
Yes	2.34 (1.30 - 4.22) 0.005	2.25 (1.10 - 4.64)	**0.027**
**Recent Antibiotic Use**			
No	1.00	1.00	–
Yes	1.58 (0.93 - 2.68) 0.092	2.09 (1.06 - 4.11)	**0.033**
**Diabetes Mellitus**			
No	1.00	1.00	–
Yes	1.82 (0.74 - 4.48) 0.193	1.94 (0.62 - 6.05)	0.253
**Education Level**			
Tertiary	1.00	1.00	–
None	1.37 (0.46 - 4.05) 0.573	1.22 (0.35 - 4.27)	0.751
Primary	2.02 (0.86 - 4.71) 0.105	1.46 (0.53 - 4.01)	0.464
Secondary	1.44 (0.58 - 3.53) 0.431	0.81 (0.28 - 2.35)	0.696
**Treatments for Vag. Inf.**			
None	1.00	1.00	–
Once	1.56 (0.76 - 3.19) 0.222	1.24 (0.51 - 3.05)	0.637
At least twice	2.66 (1.45 - 4.87) 0.001	0.93 (0.40 - 2.14)	0.865

aOR: Adjusted Odds Ratio; CI: Confidence Interval. The multivariate model included all variables with a crude p-value < 0.2.

## Discussion

In this study, 23.6% of women presenting with abnormal vaginal discharge at MRRH had *non- albicans Candida* (NAC). This indicates that nearly one in four symptomatic women in this clinical setting had NAC isolated, underscoring its epidemiological importance in the local burden of vulvovaginal candidiasis (VVC). This finding is consistent with the broader shift in VVC etiology, in which NAC species are increasingly recognized as important contributors to disease worldwide [17].

Within the African context, the prevalence observed in our study is both comparable to and different from that reported elsewhere, likely reflecting variation in study populations, diagnostic methods, and reporting denominators. For example, our estimate appears lower than the 45.0% reported in Nigeria [18] and the 41.4% reported in Ethiopia [19]. However, those studies reported NAC as a proportion of *Candida*-positive isolates rather than as a prevalence among all symptomatic women screened. This methodological distinction is important when interpreting findings across studies. In contrast, our estimate reflects the proportion of all enrolled symptomatic women with abnormal vaginal discharge who had NAC isolated.

Our result is closely aligned with the pooled estimate of 24.1% from a meta-analysis of studies in sub-Saharan Africa [16], suggesting that the pattern observed at MRRH is broadly consistent with regional epidemiology. Other African studies, including those from Cameroon and Egypt, also support a substantial burden of NAC in women with vulvovaginal symptoms [20,21]. Conversely, the prevalence in our study is higher than the 9.5% reported in Namibia, which may reflect differences in syndromic definitions, patient characteristics, and laboratory procedures [20].

Globally, the prevalence at MRRH is lower than the high NAC proportions reported from some Asian settings, where NAC has accounted for more than 60% of *Candida* isolates in some cohorts [22–24]. It is also lower than the approximately 50% reported among women with recurrent vulvovaginal symptoms in Europe and the United States [25]. This difference is not unexpected, because our study included symptomatic women with abnormal vaginal discharge in routine gynecologic care and was not restricted to women with recurrent VVC, although a substantial proportion reported previous vaginal infections. Overall, these comparisons suggest that NAC constitutes an important component of vulvovaginal *Candida* epidemiology, although direct comparison across studies should always take into account the underlying study population, diagnostic approach, and denominator used.

The multivariable analysis identified several factors independently associated with NAC, namely having two or more sexual partners, HIV-positive status, a history of recurrent vaginal infections, contraceptive use, vaginal douching, and recent antibiotic use. These findings suggest that NAC occurs within a network of interrelated behavioral and clinical factors that may alter vaginal ecology or host susceptibility.

The strong association between having two or more sexual partners and NAC suggests that sexual behavior may influence the occurrence of NAC in this setting. Although VVC is not generally classified as a sexually transmitted infection, previous studies have reported associations between candidiasis and sexual practices or number of sexual partners [26,27]. A plausible explanation is that sexual intercourse may disturb the vaginal microenvironment through transient pH changes and local mucosal irritation, thereby facilitating *Candida* overgrowth [28]. However, because of the cross-sectional design, this finding should be interpreted as a correlation in this setting rather than evidence of direct transmission. In addition, the observed association may still be influenced by residual confounding from unmeasured behaviors, as also suggested in other studies [29,30].

HIV-positive status was also strongly associated with NAC, which is consistent with literature showing increased candidal colonization and symptomatic infection among immunocompromised women [29,31]. The most likely explanation is impaired cell-mediated immunity, especially reduced CD4 + T-cell function, which weakens mucosal defense against fungal organisms [31]. HIV-related immune dysfunction therefore provides a biologically plausible explanation for the higher likelihood of NAC in this group. Although some studies have reported that HIV status may not necessarily alter symptom severity, they still support increased susceptibility to candidal infection in affected women [29].

A history of recurrent vaginal infections was another important correlate of NAC. This finding is in agreement with prior studies showing that NAC species are frequently identified among women with recurrent vulvovaginal symptoms [17,32]. However, the present data do not allow determination of temporality. It is therefore more appropriate to interpret recurrent vaginal infections as a marker associated with NAC rather than to infer a direct causal pathway.

Contraceptive use was independently associated with NAC, consistent with previous studies linking hormonal contraceptive exposure to increased occurrence of VVC [33,34]. Hormonal influences, especially estrogen-related changes in vaginal epithelial glycogen, may contribute to a microenvironment favorable for fungal growth [35]. Nevertheless, the literature remains inconsistent, and the magnitude or direction of association may differ by contraceptive type, formulation, and duration of use [36]. Since all contraceptive methods were grouped into a single variable in the present study, this association should be interpreted cautiously.

Vaginal douching was associated with more than twice the odds of NAC. This finding is consistent with evidence that douching may disrupt the protective vaginal microbiota and alter the vaginal environment in ways that favor fungal overgrowth [37,38]. The loss of protective Lactobacillus species and the chemical effects of douching products may both contribute to this association. However, our study measured douching as a binary variable and did not capture the frequency, timing, or type of substance used, factors that may further influence risk.

Recent antibiotic use was also significantly associated with NAC. This is consistent with established evidence that antibiotic exposure can disrupt the normal vaginal flora, particularly Lactobacillus species, thereby reducing colonization resistance and facilitating *Candida* proliferation [18,38,39]. Because antibiotic use is common in routine clinical care, this finding has practical implications and supports the need for more careful antimicrobial use, especially among women with recurrent vulvovaginal symptoms.

Taken together, these findings suggest that NAC in this setting is associated with overlapping behavioral and clinical factors rather than a single isolated exposure. Women with HIV infection, recurrent vaginal symptoms, recent antibiotic exposure, vaginal douching practices, contraceptive use, or multiple sexual partners may therefore represent important groups for focused counselling and closer clinical evaluation. Our findings should not be interpreted as proving vulvovaginal candidiasis in all culture-positive women, because clinical, microscopic, and pH-based exclusion of alternative causes of discharge was not performed.

### Strengths and limitations of the study

This study has several strengths. Consecutive recruitment of 314 symptomatic women reduced selection bias and strengthened the internal validity of the findings. The use of culture, germ tube testing, and CHROMagar provided a standardized phenotypic approach to Candida identification, while multivariable logistic regression enabled adjustment for multiple covariates when examining factors associated with presumptive non-albicans Candida (NAC).

Some limitations should be acknowledged. The cross-sectional design does not allow causal inference or clear temporal interpretation of the observed associations. The outcome reflects presumptive NAC isolation among women presenting with abnormal vaginal discharge rather than a definitive diagnosis of vulvovaginal candidiasis, because wet mount/KOH microscopy, vaginal pH testing, and systematic exclusion of alternative infectious causes were not performed. Species identification relied on phenotypic methods alone and was not confirmed using biochemical or molecular techniques; therefore, misclassification of closely related species and under-detection of mixed Candida infections remain possible. Antifungal susceptibility testing was not performed, so the findings cannot be interpreted in relation to antifungal resistance. The relatively limited number of presumptive NAC events may also have increased the risk of model overfitting, and some adjusted estimates should therefore be interpreted with caution.

## Conclusions and recommendations

*Non-albicans Candida* was commonly isolated among symptomatic women presenting with abnormal vaginal discharge at Mubende Regional Referral Hospital. Multiple sexual partners, recurrent vaginal infections, HIV-positive status, contraceptive use, vaginal douching, and recent antibiotic use were independently associated with presumptive non-albicans Candida. These findings should be interpreted as clinic-based associations rather than evidence of sexual transmission or direct causality. Even so, they point to practical opportunities to improve care, including strengthened syndromic evaluation, focused counselling on modifiable behaviors, more careful antibiotic use, and closer assessment of women with recurrent or persistent symptoms. Further multicenter prospective studies using broader diagnostic approaches, exclusion of alternative causes of vaginal discharge, and confirmatory species identification are needed to clarify the burden and correlates of non-albicans Candida in similar settings*.*

## Supporting information

S1 Data Dataset used for the analysis of non-albicans Candida isolation and associated factors among symptomatic women presenting with abnormal vaginal discharge at Mubende Regional Referral Hospital, Uganda. (XLSX)
